# Automatic Sleep-Arousal Detection with Single-Lead EEG Using Stacking Ensemble Learning

**DOI:** 10.3390/s21186049

**Published:** 2021-09-09

**Authors:** Ying-Ren Chien, Cheng-Hsuan Wu, Hen-Wai Tsao

**Affiliations:** 1 Department of Electrical Engineering, National Ilan University, Yilan 26047, Taiwan; 2 Graduate Institute of Electronics Engineering, College of Electrical Engineering and Computer Science, National Taiwan University, Taipei 10617, Taiwan; danny84125@gmail.com; 3 Graduate Institute of Communication Engineering, College of Electrical Engineering and Computer Science, National Taiwan University, Taipei 10617, Taiwan; tsaohw@ntu.edu.tw

**Keywords:** arousal, convolutional neural network (CNN), ensemble learning, electroencephalography (EEG), meta-classifier, polysomnography (PSG), recurrent neural network (RNN)

## Abstract

Poor-quality sleep substantially diminishes the overall quality of life. It has been shown that sleep arousal serves as a good indicator for scoring sleep quality. However, patients are conventionally asked to perform overnight polysomnography tests to collect their physiological data, which are used for the manual judging of sleep arousals. Even worse, not only is this process time-consuming and cumbersome, the judgment of sleep-arousal events is subjective and differs widely from expert to expert. Therefore, this work focuses on designing an automatic sleep-arousal detector that necessitates only a single-lead electroencephalogram signal. Based on the stacking ensemble learning framework, the automatic sleep-arousal detector adopts a meta-classifier that stacks four sub-models: one-dimensional convolutional neural networks, recurrent neural networks, merged convolutional and recurrent networks, and random forest classifiers. This meta-classifier exploits both advantages from deep learning networks and conventional machine learning algorithms to enhance its performance. The embedded information for discriminating the sleep-arousals is extracted from waveform sequences, spectrum characteristics, and expert-defined statistics in single-lead EEG signals. Its effectiveness is evaluated using an open-accessed database, which comprises polysomnograms of 994 individuals, provided by PhysioNet. The improvement of the stacking ensemble learning over a single sub-model was up to 9.29%, 7.79%, 11.03%, 8.61% and 9.04%, respectively, in terms of specificity, sensitivity, precision, accuracy, and area under the receiver operating characteristic curve.

## 1. Introduction

Poor-quality sleep negatively affects work performance [1] as well as emotional states [2,3]. A common measure of poor-quality sleep is sleep arousals [4]. The American Academy of Sleep Medicine (AASM) defines electroencephalographic arousal as [5] “An abrupt shift in electroencephalogram frequency, including alpha, theta, and/or frequencies greater than 16 Hz, lasting at least 3 s and with at least 10 s of previous stable sleep.” Polysomnography (PSG) monitors a subject’s body functions during sleep, including brain activity as measured by electroencephalogram (EEG), eye movements as measured by electrocardiography (EOG), muscle activity or skeletal muscle activation as measured by electromyography (EMG), heart rhythm as measured by electrocardiogram (ECG), respiration flow, patient movements, and arterial oxyhemoglobin saturation (SaO2). Training set records are annotated with a patient’s sleep stages over time and any arousals experienced. While effective for analysis, PSG signals are time-consuming and cumbersome to collect. They also necessitate many cables with contact sensors, which can cause discomfort for the subject, thus influencing the results.

This research aims at designing an automatic sleep-arousal detector that requires a single-lead EEG signal only, which can be collected easily using a headphone-like device [6] so that the discomfort for the subject can be reduced. The objective is to exploit a stacking ensemble learning framework such that the sleep-arousal-related information can be extracted from the time-domain, frequency-domain, and expert-defined statistics associated with the single-lead EEG signals. Our main contributions are as follows: First, we leveraged a stacking ensemble learning framework, which is comprised of a one-dimensional (1D) convolutional neural network (CNN), a recurrent neural network (RNN), and random forest classifiers, to further enhance its specificity (85.75%), sensitivity (82.67%), precision (83.92%), and accuracy (84.92%). This approach differs from conventional ensemble learning in its blend of deep learning method with machine method. Second, we propose using band power features as inputs to learn variation and time-dependency using multi-layer long short-term memory (LSTM) networks. In addition, we exploited multitaper spectral analysis to alleviate the spectral leakage in the calculation of band power features.

The remainder of this paper is organized as follows: Section 2 reviews related work. Section 3 presents the proposed sleep-arousal detector while Section 4 presents experimental results to verify its efficacy. Conclusions are drawn in Section 5.

## 2. Related Work

Several previous studies have adopted a single EEG signal to detect the sleep-arousal events [7,8,9,10,11]. In [7], the authors proposed using a single-lead sleep EEG (C3-A2) to build features based on time-frequency analysis, which were then fed to a support vector machine (SVM) classifier. Among nine penitents with respiratory sleep disorders, the sensitivity and specificity for the training sets were 87.92% and 95.56%, respectively; values for the testing sets were 75.26% and 93.08%, respectively. Note that we believe their SVM classifier would overfit due to the insufficient records of the training dataset (three subjects). The authors then extended their work with additional features, including power variations in EEG frequency bands [8]. However, the resulting sensitivity and specificity for the testing sets were, respectively, 79.06% and 89.95%, which are inferior to those of their previous work. In [9], the authors adopted a curious extreme learning machine (C-ELM) algorithm to train a single-hidden-layer feed-forward neural network. A total of 22 features were extracted from the signals measured from one central EEG channel. The resulting averaged area under the receiver operating characteristic curve (AUROC) and the accuracies were 85% and 79%, respectively; however, their dataset is not publicly accessible. Note that there were only 144 positive data pairs (i.e., ‘arousal-detected’ labels) among a total of 7680 data pairs. In [10], the authors applied continuous wavelet transform (CWT) to a single-channel EEG signal; two features were then extracted from the scalogram, which was the squared magnitude of the CWT, as the input of the SVM classifier. The overall values for sensitivity, specificity, accuracy, and precision were all greater than 94%; however, these results were calculated from EEG data obtained from only five patients. Thus, consistency could vary as the number of patient records increases. In [11], the authors proposed using deep transfer learning for improving single-lead EEG arousal detection; they trained a model based on multi-channel PSG data and transferred it to a target domain that contained only single-lead EEG signals. With sophisticated fine-tuning, the difference between single-lead EEG and multi-channel PSG was insignificant. The resulting performance in terms of sensitivity and precision was 71.00% and 67.60%, respectively. However, mismatch issues between source and target domains must be solved in such a transfer learning approach.

From among the 13 types of raw PSG signals, some studies have selected more than one channel as the input for arousal detection. In [12], the authors utilized two EEG channels and one EMG signal to detect the EEG arousal events using artificial neural networks (ANNs). They defined 39 features from EEG signals and four contextual features. The resulting sensitivity and specificity were respectively 86% and 76%. The corresponding AUROC was 81.10%. In [13], the authors selected the eight most representative channels from the PSG signals. The pre-processed signals concerning those eight channels were fed into multiple CNNs to make individual decisions. These preliminary decisions were then fed into a random forest algorithm to make the final decision, with a resulting AUROC of 95.30%. In [14], the authors used PSG records as inputs to sequence-to-sequence deep neural networks with bi-directional LSTM and exponential linear unit networks. They used three models, each comprising bi-directional LSTM networks with different input signals. The final classification results were determined by fusing the three models with an equal-weight averaging strategy: the resulting AUROC was 95%. In [15], the authors used PSG signals to develop a feature matrix comprising EEG, EMG, leg movement, and respiratory and heart rate features. The K-nearest neighbors (KNN) classifier was implemented to detect sleep-arousal events, and the resulting average sensitivity, specificity, and accuracy were 79.0%, 95.5%, and 93.6%, respectively. However, the average performance was obtained from only nine patients. Note that the worst performance reported for sensitivity, specificity, and accuracy was only 67.50%, 97.10%, and 86.50%, respectively. In addition, approaches that rely on complete PSG signals may limit the promotion of a consumer-grade sleep-arousal detector.

In [16], the authors evaluated all PSG signals and selected six channels as input; two of these belong to the EEG signal. They constructed a deep-learning architecture comprising a convolutional-residual network and a positional embedding multi-head attention mechanism. Although overall AUROC was 84.88%, the resulting AUROC with only one EEG channel was less than 76.20%. In [17], the authors proposed 27 features (i.e., 14 EEG/EOG, seven ECG, and six EMG features) as the input for a random forest classifier, with a resulting AUROC of 84.70%. In [18], the authors applied 13 raw PSG signals to a 13-layer CNN network for an AUROC of 48.6%. This implies that some expert-defined features might help to improve classification performance. However, when the authors utilized 68 features in a 68×21 feature matrix for a three-layer neural network, the resulting AUPRC was 42.00%. In [19], the authors proposed using the scattering transform for raw PSG signals. For each signal, 36 coefficients were obtained and fed into a sequence learning machine, which was implemented by a three-layer LSTM network. Among each layer, batch-normalization blocks were used to obtain better training results, and the resulting AUROC was 88.00%. An extension of this work using a bi-directional LSTM (Bi-LSTM) to improve performance has been reported in [20]. In [21], the authors used 12 out of 13 PSG signals, excluding ECG signals, in a dense recurrent CNN, which combined a dense CNN with LSTM networks—the resulting AUROC was 93.10%.

This diverse range of approaches makes a fair and unbiased comparison of existing studies difficult. As pointed out in [9], the applied datasets differ or are not publicly accessible and physiological signals and performance metrics vary. The underlying issue is the lack of an established standard for sleep-arousal detection.

## 3. Proposed Sleep Arousal Detector

**Figure 1:**
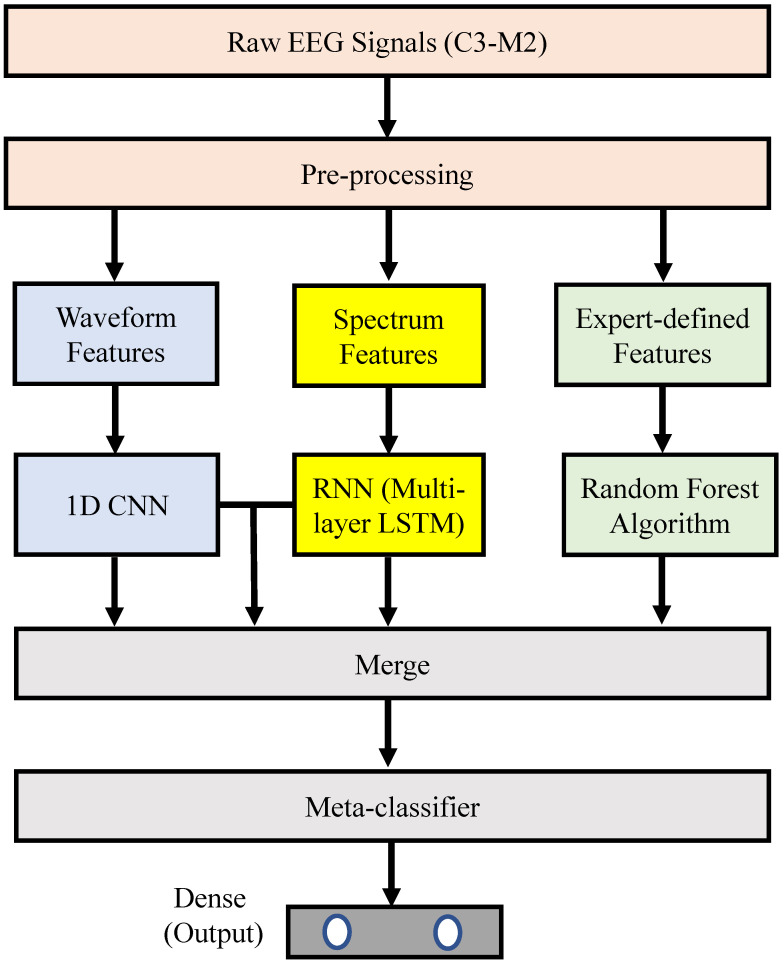
Block diagram of the proposed stacking ensemble learning approach for sleep-arousal detection.

Figure 1 illustrates the proposed sleep-arousal detector. It consists of three main parts: (1) a pre-processing method for the raw single-lead EEG signal (i.e., the C3-M2 signal); (2) a base classifier, which consists of four sub-models (namely, a 1D CNN, an RNN, a random forest classifier, and a pre-merged CNN and RNN); and (3) a meta classifier. The details of each part are described in the following.

### 3.1. Dataset and Its Pre-Processing

**Figure 2:**
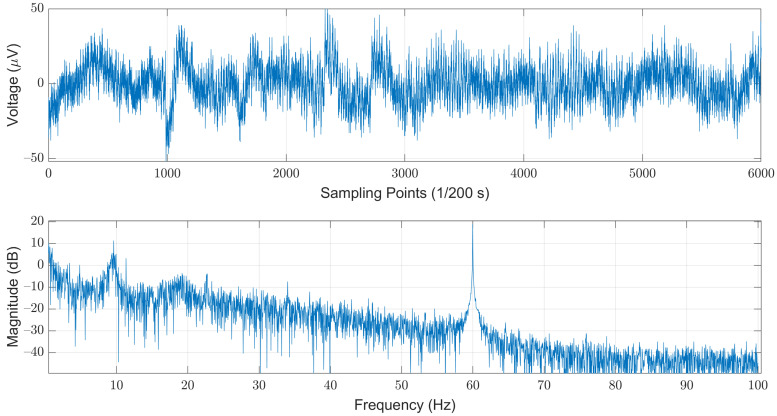
An example of the original EEG signals illustrated in time (**top**) and frequency (**bottom**) domains.

We selected the open dataset provided by the Computational Clinical Neurophysiology Laboratory (CCNL) at Massachusetts General Hospital (MGH), which was adopted in the PhysioNet/Computing in Cardiology Challenge (CinC) 2018 [22]. The dataset is comprised of 994 records of complete PSG data as well as corresponding labels. The PSG data were obtained from 13 channels, which include a six-lead EEG signal (F3-M2, F4-M1, C3-M2, C4-M1, O1-M2, and O2-M1), a three-lead EMG (ABD, CHEST, and Chin1-Chin2), single-lead ECG, SaO2, Airflow, and EOG (E1-M2). The length of each record varies from four to eight hours. The sampling rate for each piece of data is 200 Hz, and each sampled value was stored in a 16-bit number system. The corresponding labels for each sampled data are Boolean values, where “0” and “1” denote “normal” and “sleep-arousal” events, respectively. Among these 13 channels, we choose the C3-M2 single-lead EEG signal as our sole data source. Note that it has been pointed out that arousals can be scored from the central EEG [23]. In addition, most of the related works that used single-channel EEG are also adopting left central EEG (C3). The steps of data pre-processing included noise filtering, segmentation, and re-labeling for each segment. As illustrated in Figure 2, the original EEG signals are contaminated by the powerline-induced noises at the mains voltages. In this example, we could easily observe strong powerline-induced noises at 60 Hz. Therefore, the first step of the data pre-processing is to remove these noises to obtain a cleaner EEG signal.

**Figure 3:**
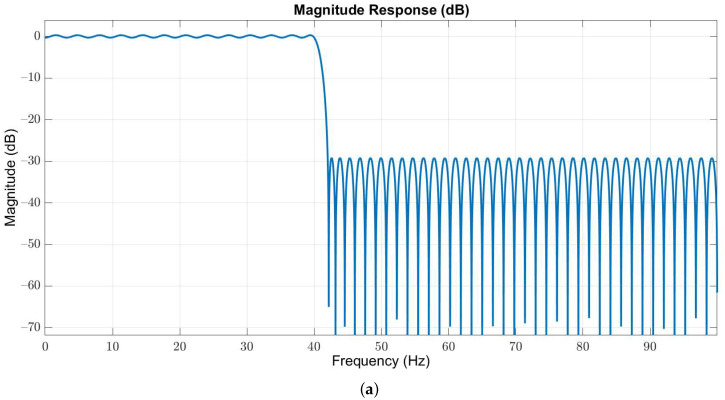

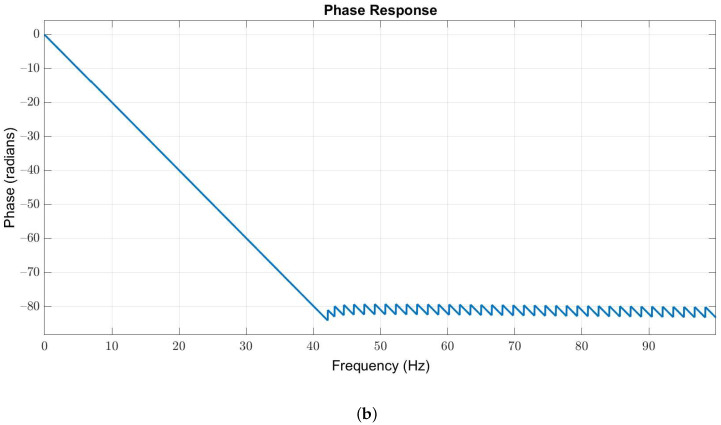
(**a**) Magnitude and (**b**) phase responses of the low-pass filter with passband and stopband edges at 40 Hz and 42 Hz, respectively.

Owing to the useful frequency information of EEG signals is below 40 Hz, we used a 127-order low-pass Butterworth filter with passband and stopband edges at 40 Hz and 42 Hz, respectively, to obtain the in-band EEG signals. The magnitude and phase responses are illustrated in Figure 3a,b, respectively.

**Figure 4:**
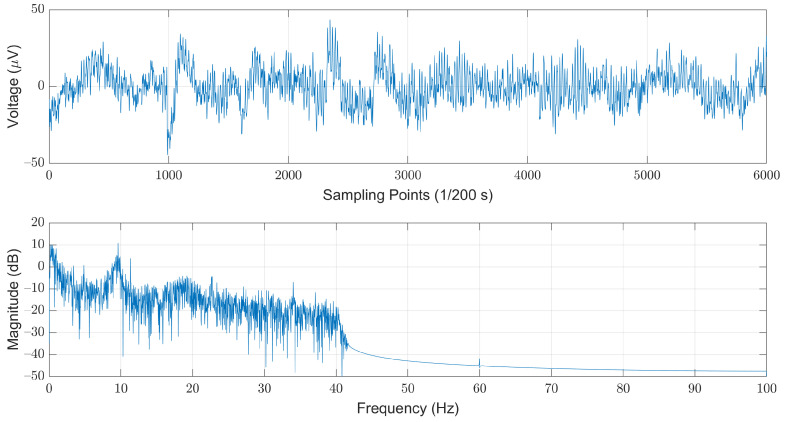
The low-pass filtered EEG signals illustrated in time (**top**) and frequency (**bottom**) domains.

As shown in Figure 4, this filter can effectively remove most of the interference induced by the powerline while maintaining in-band EEG signals with neglectable distortions. For each record, we first discarded the first and the last hours, and then divided the remaining records into 30-second segments. The choice of the length of each segment refers to the AASM guidelines that suggest scoring arousals in a 30-s window [24]. Note that the last segment was discarded if the length was less than 30 s.

**Figure 5:**
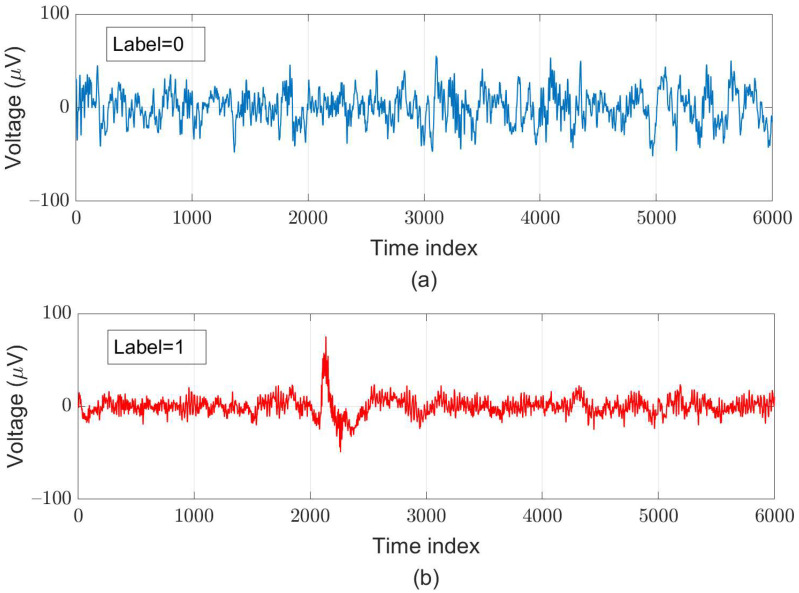
Examples of (**a**) normal (label = 0) and (**b**) arousal (label = 1) segments each with a 30-s length obtained at the C3-M2 channel.

For supervised learning, we assign the ground truth for each segment according to the original ground truth for each sample and the AASM’s definition of sleep arousal. After pre-processing, we created 119,983 data pairs, in which around 40% of these pairs are labeled as “sleep-arousal” events (47,993 data pairs) and the other 71,990 data pairs are labeled as “normal” events. Among these segments, 22,328 pairs (about 20% of the total segments) served as testing data; for five-fold cross-validation, we used 78,124 pairs as training data and adopted 19,531 pairs as validation data. Examples of a normal segment and an arousal segment are illustrated in Figure 5.

### 3.2. Feature Extraction

We applied 1D CNN networks to the extraction of embedded features in the waveform for each segment of length 6000×1. In addition to the waveform features, we adopted 29×8 band power features and 1×42 expert-defined features.

#### 3.2.1. Band Power Features

It has been reported that changes of EEG power across the frequency band reveal information about arousal detection [25]. This prompted us to apply an RNN to investigate the significance of band-power patterns. We considered eight features derived from the frequency domain, which comprised the power of EEG signals in the delta-band (0–4 Hz), theta-band (4–7 Hz), alpha-band (8–12 Hz), and beta-band (14–30 Hz), as well as the full band (0–40 Hz) for each segment. Moreover, we considered three power ratios to account for variations among bands: (1) delta to theta; (2) theta to alpha; and (3) delta to alpha. Conventionally, a periodogram is applied to estimate power spectral density to calculate the signal power within a specific frequency band. However, due to the highly-oscillatory dynamics exhibited by biomedical signals, a good balance between the bias and variance of the spectral estimation problem cannot be obtained using a periodogram. Instead, we applied multitaper spectral analysis for its advantages of both low variance and high-frequency resolution [26,27]. In [28], the authors provided a C-subroutine to calculate the coefficients of the discrete prolate spheroidal sequences (DPSS) or Slepian sequences. Multitaper spectral estimation for the *m*-th segment $x_{m}$ can be expressed as follows:

(1) $$\begin{array}{c} \hat{S}\left(k\right)=\frac{1}{N_{L}}\sum_{i=1}^{N_{L}}{\hat{S}}^{\left(i\right)}\left(k\right) \end{array}$$

with

(2) $$\begin{array}{c} {\hat{S}}^{\left(i\right)}\left(k\right)={\left|\sum_{n=0}^{N_{b}-1}x_{m}\left(n\right)\cdot h^{\left(i\right)}\left(n\right)e^{-j\frac{2\pi nk}{N_{b}}}\right|}^{2}, \end{array}$$

where $k=0,1,\ldots ,N_{b}-1$ is the index of the frequency bin; $x_{m}\left(n\right)$ denotes the *n*-th element of the *m*-th segment $x_{m}$, and $h^{\left(i\right)}\left(n\right)$ represents the *n*-th coefficients of the *i*-th DPSS filter $h^{\left(i\right)}\left(n\right)$. We performed spectral analysis every two seconds (400 samples) with an overlap factor of 50%. This resulted in 29 spectral analyses, with 201 frequency bins for each segment. Note that we chose the length of each DPSS taper as $N_{b}=400$ and the number of tapers as $N_{L}=8$. Finally, we estimate the power as well as the power ratio and derived the frequency feature matrix with the dimensions 29×8 for each segment.

**Figure 6:**
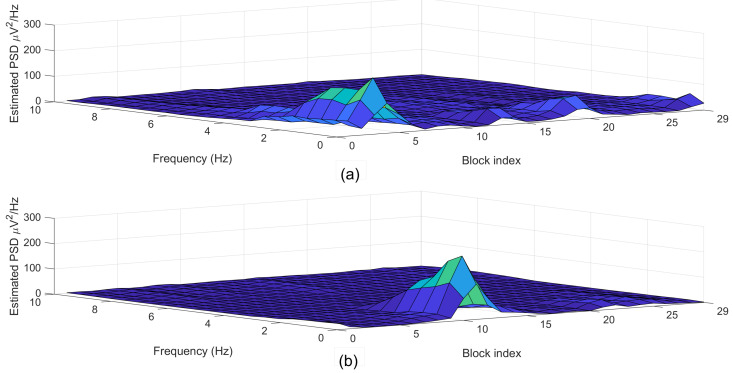
Examples of (**a**) normal (label = 0) and (**b**) arousal (label = 1) segments each with a 30-s length.

Figure 6 depicted the estimated power spectral density (PSD) diagrams that correspond to the segments plotted in Figure 5.

With the estimated PSD for each segment, we could numerically apply the composite Simpson’s or Euler–Maclaurin rule [29] to integrate the PSD function within a specific frequency range. For example, the power can be estimated for the delta band (0 to 4 Hz) by integrating the PSD within the frequency bins from the first to the ninth bins. The other band power features can be calculated in similar ways as those listed in Table 1 below. Note that the frequency resolution is 0.5 Hz and the first bin maps to the direct current (DC) gain. Therefore, the three power ratios (i.e., (1) delta to theta; (2) theta to alpha; and (3) delta to alpha) could be easily calculated.

#### 3.2.2. Expert-Defined Features

The expert-defined features were measured in both time and frequency domains. We used a 1×42 vector for each segment to represent the corresponding expert-defined features listed in Table 2.

For frequency-domain expert-defined features, we calculate the mean, minimum, standard deviation, and the 95th percentile with respect to the eight band power features. In addition, we measured the kurtosis concerning the sub-matrix of the frequency matrix. We used only the data associated with delta, theta, alpha, and sigma bands.

In addition to the mean, standard deviation, average numerical gradient, maximum, and minimum values, experts usually evaluate the Hjorth parameters [30], kurtosis, and skewness values. Hjorth reported using activity, mobility, and complexity to portray EEG signals in the time domain. The Hjorth activity $\sigma_{H}^{2}\left(x_{m}\right)$ measures the variance for the *m*-th segment $x_{m}$ and can be calculated as follows:

(3) $$\begin{array}{c} \sigma_{H}^{2}\left(x_{m}\right)=\frac{\sum_{n=0}^{N-1}{\left(x_{m}\left(n\right)-\mu_{m}\right)}^{2}}{N-1}, \end{array}$$

where $x_{m}\left(n\right)$ represents the *n*-th element of $x_{m}$ and $\mu_{m}=\frac{1}{N}\sum_{n=0}^{N-1}x_{m}\left(n\right)$ denotes the sample-mean of $x_{m}$; The Hjorth mobility $M_{H}\left(x_{m}\right)$ estimates the main frequency content for the *m*-th segment $x_{m}$ and can be expressed as follows:

(4) $$\begin{array}{c} M_{H}\left(x_{m}\right)=\sqrt{\frac{\sigma_{H}^{2}\left(\mathrm{Diff}\left(x_{m}\right)\right)}{\sigma_{H}^{2}\left(x_{m}\right)}}, \end{array}$$

where $\mathrm{Diff}\left(\cdot \right)$ is the difference operation. The Hjorth complexity $C_{H}\left(x_{m}\right)$ estimates the bandwidth associated with the *m*-th segment $x_{m}$ and can be expressed as follows:

(5) $$\begin{array}{c} C_{H}\left(x_{m}\right)=\frac{M_{H}\left(\mathrm{Diff}\left(x_{m}\right)\right)}{M_{H}\left(x_{m}\right)}. \end{array}$$

Kurtosis is an index that determines whether the data are heavy- or light-tailed relative to Gaussian-distributed data and can be calculated as follows:

(6) $$\begin{array}{c} K_{m}=\frac{\frac{1}{N}\sum_{n=0}^{N-1}{\left(x_{m}\left(n\right)-\mu_{m}\right)}^{4}}{\sigma_{m}^{4}}, \end{array}$$

with standard deviation $\sigma_{m}$ defined as follows:

(7) $$\begin{array}{c} \sigma_{m}=\sqrt{\frac{\sum_{n=0}^{N-1}{\left(x_{m}\left(n\right)-\mu_{m}\right)}^{2}}{N-1}}. \end{array}$$

For Gaussian-distributed data, the value of kurtosis is three. The corresponding distribution tends to have heavy tails for data with high kurtosis and thus exhibits more outliers. This implies that, for data with low kurtosis, there are light tails and a lack of outliers.

The skewness of data is used to evaluate the asymmetry of the probability distribution of a real-valued random variable about its mean. The skewness of the *m*-th segment $x_{m}$ can be calculated as follows:

(8) $$\begin{array}{c} S_{m}=\frac{\frac{1}{N}\sum_{n=0}^{N-1}{\left(x_{m}\left(n\right)-\mu_{m}\right)}^{3}}{\sigma_{m}^{3}}, \end{array}$$

where $\sigma_{m}$ is the sample standard deviation defined in Equation (7).

### 3.3. Stacking Ensemble Learning

For stacking ensemble learning, we adopted a meta-classifier to stack the outputs of the three sub-models as input and attempted to learn the best combinations for the input classifications in order to construct a better output classification.

#### 3.3.1. Waveform Raw Data for 1d Cnn Networks

**Figure 7:**
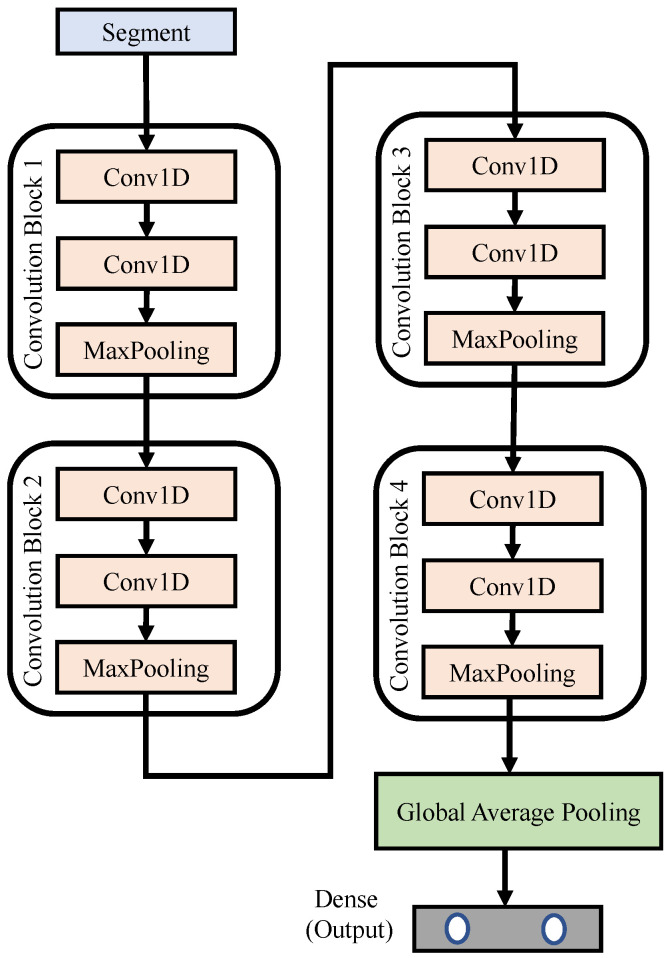
Block diagram of the proposed 1D CNN networks.

Figure 7 depicts the block diagram of the proposed 1D CNN networks, where *N* denotes the total number of segments used to train the network. The input signal was a pre-processed segment, each with a length of 6000 samples; that is, the time duration was 30 s. The proposed 1D CNN networks were composed of four convolution blocks. The details of each block can be found in Table 3. The lengths of the feature detector within each block were 50, 30, 10 and 2, respectively. This design helps the network to dig more subtle variations or features embedded in each segment. Finally, we use a global average pooling and dense layer, in which a softmax activation function is used to limit the predicted values to fall between 0 and 1. The 2×1 output vector of the dense layer predicts the possibility of “normal” and “arousal detected” events, respectively.

#### 3.3.2. Band Power Features for Rnn Networks

**Figure 8:**
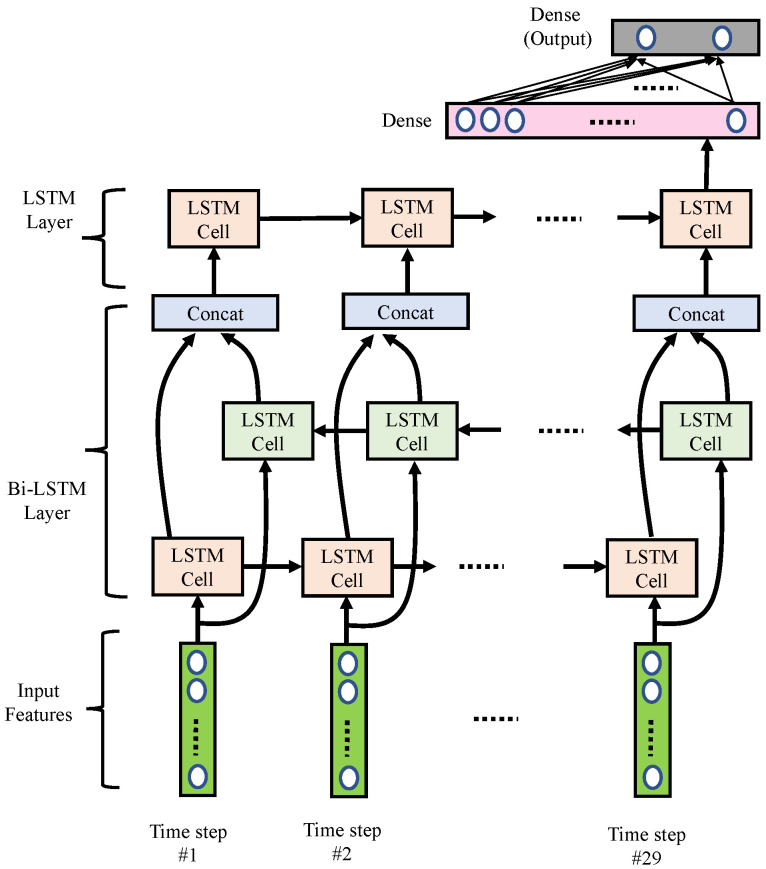
Block diagram of the proposed RNN networks.

As shown in Figure 8, we implemented the RNN using multi-layer LSTM networks containing one bi-directional LSTM layer and one uni-directional LSTM layer. Detailed parameters are listed in Table 4. This LSTM network excavates the power variation and power ratio variation for the bands calculated in the 29×8 band power features. Thus, we had 29 time-steps, with eight features for each segment. A bidirectional LSTM layer consisted of one forward LSTM and one backward LSTM, each containing 20 hidden states. Next, we concatenated the forward and backward LSTM cells with the concatenation blocks. Then, an extra uni-directional LSTM layer with a less hidden state size (i.e., ten hidden LSTM units) was used. Note that the last hidden state of the uni-directional LSTM would pass through to a dense layer with 32 nodes. Finally, a dense output layer with two nodes was used to generate a 2×1 output vector, which was used to represent the probabilities of normal and arousal classes associated with the input features.

#### 3.3.3. Expert-Defined Features for Random Forests

A random forest algorithm produces mass decision trees and is trained by performing bagging operation to combine multiple decision trees in order to achieve more stable and accurate classification results. We adopted the 1×42 expert-defined features for each segment as input information for the random forest algorithm.

### 3.4. Stacking Ensemble

**Figure 9:**
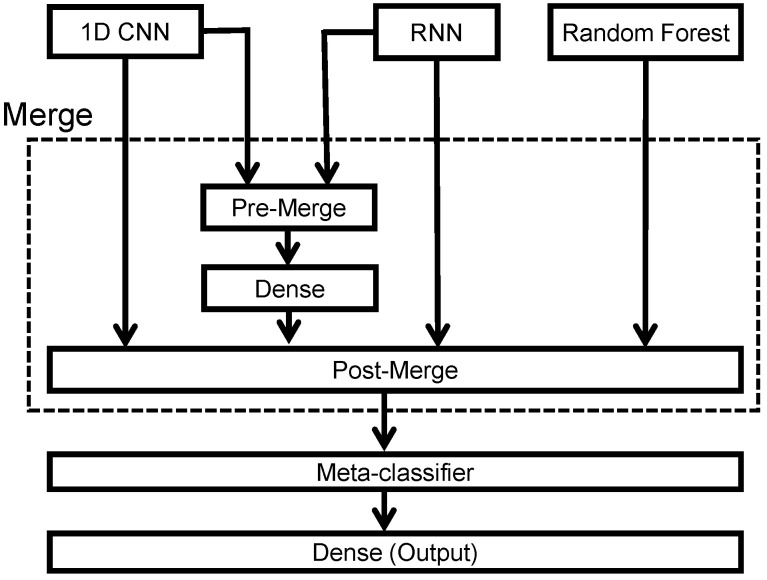
Block diagram of the stacking ensemble learning.

To leverage the specific advantages of 1D CNN networks, RNN networks, and random forest algorithms, we merged the classification outputs and the intermediate results into a meta-classifier to perform stacking ensemble learning. The block diagram of the proposed stacking ensemble learning architecture is shown in Figure 9. Note that we proposed using a pre-merge module to combine the information at the input of the dense layer in both 1D CNN and RNN networks. The merged 1D CNN and RNN networks extract complementary information that is missed for either CNN or RNN alone and aim to exploit the temporal and spatial characteristics information to achieve better classification results. Thus, the pre-merge module combined the 12×1 and 32×1 vectors into a 44×1 vector, and the post-merge module fused four 2×1 vectors to train the meta-classifier.

Our meta-classifier is trained with the logistic regression method. This is a simple linear model for a binary classification outcome (i.e., arousal detected (1) or normal (0)). Logistic regression is a statistical model that uses a logistic function to model a binary dependent variable in its basic form. For the logistic-regression-based meta-classifier, the posterior probability of a binary response variable *Y* with inputs $p={\left[1,p_{1},p_{2},\ldots ,p_{N-1},p_{N}\right]}^{T}$ and regression coefficient vector $\beta ={\left[\beta_{0},\beta_{1},\ldots ,\beta_{N}\right]}^{T}$ can be expressed as follows:

(9) $$\begin{array}{c} P_{Y}\left(y|p,\beta \right)={\left(h_{\beta }\right)}^{y}\cdot {\left(1-h_{\beta }\right)}^{1-y}, \end{array}$$

with

(10) $$\begin{array}{c} h_{\beta }=\frac{\exp\left\{p^{T}\beta \right\}}{1+\exp\left\{p^{T}\beta \right\}}, \end{array}$$

where the label *y* is either 0 or 1; *T* denotes the transpose operation; the inputs $\left(p_{2i-1},p_{2i}\right)$ for $i\in \left\{1,2,3,4\right\}$ denote the predicted probabilities for normal and arousal events for the *i*th sub-model (*N* = 8); and $\beta_{0}$ is the model intercept. The training procedure aims at finding the optimal regression coefficients β with the training data p by minimizing the log-likelihood cost function:

(11) $$\begin{array}{c} J\left(\beta \right)=\frac{-1}{M}\sum_{j=1}^{M}y_{j}\log\left(h_{\beta }\left(p_{j}\right)\right)+\left(1-y_{j}\right)\log\left(1-h_{\beta }\left(p_{j}\right)\right), \end{array}$$

where *M* is the number of training inputs and $p_{j}$ denotes the *j*th p inputs. By using the gradient descent method, we can obtain the optimal regression coefficients $\beta^{*}$. Thus, if the posterior probability $P_{Y}\left(y=1|p,\beta^{*}\right)$ is greater than 0.5, we claim that the sleep arousal is detected.

## 4. Experiments

### 4.1. Evaluation Metrics

In this work, we adopted accuracy, sensitivity, specificity, precision, and AUROC as evaluation metrics. We define these as follows:

(12) $$\begin{array}{cc} \mathrm{Accuracy} & =\frac{\mathrm{TP}+\mathrm{TN}}{\mathrm{TP}+\mathrm{FP}+\mathrm{FN}+\mathrm{TN}}. \end{array}$$

(13) $$\begin{array}{cc} \mathrm{Sensitivity} & =\frac{\mathrm{TP}}{\mathrm{TP}+\mathrm{FN}}. \end{array}$$

(14) $$\begin{array}{cc} \mathrm{Specificity} & =\frac{\mathrm{TN}}{\mathrm{FP}+\mathrm{TN}}. \end{array}$$

(15) $$\begin{array}{cc} \mathrm{Precision} & =\frac{\mathrm{TP}}{\mathrm{TP}+\mathrm{FP}}, \end{array}$$

where “TP” denotes the number of true positive results, “TN” denotes the number of true negative results, “FP” denotes the number of false positive results, and “FN” denotes the number of false negative results in the confusion matrix.

AUROC is a performance measurement for classification problems at various threshold settings. The receiver operating characteristic (ROC) curve is plotted with the TP rate against the FP rate (i.e., $1-\mathrm{Specificity}=\mathrm{FP}/\left(\mathrm{TN}+\mathrm{FP}\right)$). The higher the values of AUROC are, the better the model distinguishes between normal and sleep-arousal classes.

### 4.2. Results

#### 4.2.1. Training Results

The averaged performances with five-fold cross-validation are listed in Table 5. Note that, although the resulting performances associated with the CNN and RNN sub-models were below 80%, the meta-classifier could exploit the advantages of each sub-model to boost the overall performance.

#### 4.2.2. Testing Results

For the 22,328 segments, the resulting performance for each sub-model and the meta-classifier are shown in Table 6. As expected, stacking ensemble learning improved performance. This may infer that our model does not over-fit during training phases.

Table 7 compares this work with state-of-the-art methods that use single-lead EEG signals to detect the sleep arousal events. Note that the results reported in [7,10] are not reliable because the number of patients is too low to confirm reliability. From the reported result in [7], we even believe their SVM classifier would overfit with the few records of the training dataset (three subjects). However, compared with [11], which had 1500 patients in their experiments, the proposed method exhibited significant improvements in sensitivity and precision.

## 5. Conclusions

Collecting complete PSG signals to detect sleep-arousal hinders the development of a consumer-grade portable sleep arousal detector. Therefore, we propose a novel sleep-arousal detection method that requires only single-lead EEG signals based on the stacking ensemble learning framework. The meta-classifier stacks four sub-models: (1) 1D CNN; (2) RNN (multi-layer LSTM); (3) merged 1D CNN and RNN; and (4) random forest algorithm. First, the 1D CNN network extracts the embedded features in the time-domain waveform. Second, the RNN learns the temporal dependence in the band power and power ratio features. Third, the merged 1D CNN and RNN networks extract complementary missing information for either CNN or RNN alone. Finally, the random forest algorithm exploits the expert-defined features calculated from both time and frequency domains. We verified the effectiveness of the proposed method using the open-accessed database compiled in [22]. The improvements of the meta-classifier over any sub-model can be up to 9.29%, 7.79%, 11.03%, 8.61%, and 9.04%, respectively, in terms of specificity, sensitivity, precision, accuracy, and AUROC. However, because the statistical features were extracted from each segment of 30 s, the time resolution to discriminate sleep-arousals is 30 s. Besides, we have sacrificed the accuracy for simplicity such that the implementation of the portable sleep-arousal detector becomes possible. In future work, we aim to fuse other physiological signals, such as EMG, to enhance the resulting accuracy of the detector while considering the comfortability when collecting those physiological data.

## Figures and Tables

**Table 1:** Band power features and the corresponding frequency bins.

Band	Frequency Ranges (Hz)	Frequency Bins
Delta	0 to 4	1 to 9
Theta	4 to 8	10 to 17
Alpha	8 to 12	18 to 25
Beta	14 to 30	30 to 61
Full	0 to 40	1 to 81

**Table 2:** Expert-defined features.

Domain	Feature	Number of Features
Time	Averaged numerical gradient	1
	Kurtosis	1
	Hjorth parameters	3
	Skewness	1
Frequency	Minimum *	8
	Mean *	8
	Standard deviation *	8
	95th percentile *	8
	Kurtosis ${}^{†}$	4

Note: * denotes the features are calculated from the four sub-bands, three band-power ratios, and one full-band; ${}^{†}$ denotes the features are calculated from the four sub-bands.

**Table 3:** Detailed settings of the proposed 1D CNN networks.

Layer	Filter Size	Activation Function	Number of Trainable Parameters	Output Shape (x, y, z)
Input	-	-	0	(N, 6000, 1)
Conv1D${}_{1}$	20@50×1	ReLU	1020	(N, 5951, 20)
Conv1D${}_{2}$	20@50×1	ReLU	20,020	(N, 5902, 20)
Maxpooling${}_{1}$	2×1	-	0	(N, 2951, 20)
Conv1D${}_{3}$	20@30×1	ReLU	12,020	(N, 2922, 20)
Conv1D${}_{4}$	24@30×1	ReLU	14,424	(N, 2893, 24)
Maxpooling${}_{2}$	2×1	-	0	(N, 1446, 24)
Conv1D${}_{5}$	12@10×1	ReLU	5784	(N, 1437, 12)
Conv1D${}_{6}$	12@10×1	ReLU	2892	(N, 1428, 12)
Maxpooling${}_{3}$	2×1	-	0	(N, 714, 12)
Conv1D${}_{7}$	12@2×1	ReLU	300	(N, 713, 12)
Conv1D${}_{8}$	12@2×1	ReLU	300	(N, 712, 12)
Maxpooling${}_{4}$	2×1	-	0	(N, 356, 12)
Global Average Pooling	-	-	0	(N, 12, 1)
Dense (output)	-	Softmax	26	(N, 2, 1)

**Table 4:** Detailed settings of the proposed RNN networks.

Layer	Number of Hidden State Size	Activation Function	Number of Trainable Parameters	Output Shape (x, y, z)
Input	-	-	0	(N, 29, 8)
Bi-directional LSTM (Bi-LSTM)	20	-	4640	(N, 29, 40)
Uni-directional LSTM	10	-	2040	(N, 10, 1)
Dense	-	ReLU	352	(N, 2, 1)
Dense (output)	-	Softmax	66	(N, 2, 1)

**Table 5:** Training results for the proposed sleep-arousal detector.

Model	CNN	RNN	Random Forest	Pre-Merge	Meta-Classifier
Specificity (%)	77.98	75.60	83.24	78.03	85.75
Sensitivity (%)	78.38	73.73	77.85	80.14	82.67
Precision (%)	72.46	68.85	77.03	76.67	83.92
Accuracy (%)	78.15	74.81	80.98	79.03	84.92

**Table 6:** Testing results for the proposed sleep-arousal detector.

Model	CNN	RNN	Random Forest	Pre-Merge	Meta-Classifier
Specificity (%)	77.48	75.35	82.14	77.43	84.64
Sensitivity (%)	78.34	72.31	77.51	78.60	80.10
Precision (%)	73.36	69.53	77.21	76.79	80.56
Accuracy (%)	77.86	74.02	80.11	78.00	82.63
AUROC (%)	79.97	74.97	81.54	80.23	84.01

**Table 7:** Comparisons with state-of-the-art methods that used a single-lead EEG only.

Work	Year	Method	Number of Patients	Results
[7]	2005	SVM	9	sensitivity: 75.26% specificity: 95.56%
[8]	2007	SVM	20	sensitivity: 79.06% specificity: 89.95%
[9]	2015	C-ELM	50	accuracy: 79.00% AUROC: 85.00%
[10]	2019	SVM	5	sensitivity: 94.67% specificity: 99.33% precision: 97.93% accuracy: 98.20%
[11]	2020	2D CNN	1500	sensitivity: 67.60% precision: 71.00%
This work	2021	Stacking Ensemble Learning	994	sensitivity: 84.64% specificity: 80.10% precision: 80.56% accuracy: 82.63% AUROC: 84.01%

## Data Availability

Please see the link https://physionet.org/content/challenge-2018/1.0.0/ (accessed on 1 August 2019) for accessing the open-accessed data we used in this work. Anyone can access the files, as long as they conform to the terms of the Open Data Commons Attribution License v1.0.
